# Locked nailing for the treatment of displaced articular fractures of the calcaneus: description of a new procedure with calcanail^®^

**DOI:** 10.1007/s00590-012-0968-1

**Published:** 2012-03-13

**Authors:** Mario Goldzak, Thomas Mittlmeier, Patrick Simon

**Affiliations:** 1 Clinique de l’Union, 31240 Saint Jean, France; 2 Medizinische Facultät, Chirurgischen Klinik und Poliklinik des Univeristät Rostock, PF 10 08 88, 18055 Rostock, Germany; 3 Centre hospitalier Saint Joseph Saint Luc, 20 quai Claude Bernard, 69365 Lyon Cedex, France

**Keywords:** Calcaneus, Fracture fixation, Intramedullary nailing

## Abstract

Although open reduction and internal fixation is considered the best method for treating displaced articular fractures of the calcaneus, lateral approach is at high risk for wound healing complications. For this reason, the authors developed a posterior approach and a new implant to perform both intrafocal reduction and internal fixation. The aim of this technical note is to describe this method of treatment for displaced articular fractures of the calcaneus, which offered the following advantages: (a) the creation of a working channel that provides also a significant bone autograft, (b) the intrafocal reduction of the displaced articular surface, (c) the insertion of a locking nail that maintains the reduced articular surface at the right height, (d) the possibility to switch from an ORIF to a reconstruction arthrodesis with the same approach and instrumentation in case of severely damaged posterior facet.

## Introduction

Open reduction and internal fixation is considered the best method for treating displaced articular fractures of the calcaneus [1, 2]. The anatomic situation of the fracture line and the displacement of the main fragment are precised by preoperative CT scan [3]. The reduction of the osteochondral depressed fragments is usually achieved by a lateral approach and a more or less extensive plate is placed on the lateral face of the calcaneus. However, lateral approach of the calcaneus is at risk of delayed wound healing, skin necrosis, or infection. The overall wound complication rate was 33% in the Abidi retrospective study [4] and 16% in the Howard prospective randomized trial [5]. Rates of up to 5% of deep infection have been reported [5]. Although outcomes scores tend to support internal fixation for articular calcaneal fractures, the high complications rate may limit surgical indications [5].

Other operative procedures have been proposed, including two stages procedures [6], medial approaches, or percutaneous fixation [7]. These procedures are demanding and actually reserved to experienced surgeons.

For these reasons, we developed a posterior approach and a new implant to perform both intrafocal reduction and internal fixation. The aim of this technical note is to describe this method of treatment for displaced articular fractures of the calcaneus.

## Procedure

The patient is placed on a standard operating table in a lateral position, the foot outside of the table: so lateral view and axial retrotibial views are easily obtained.

**Fig. 1:**
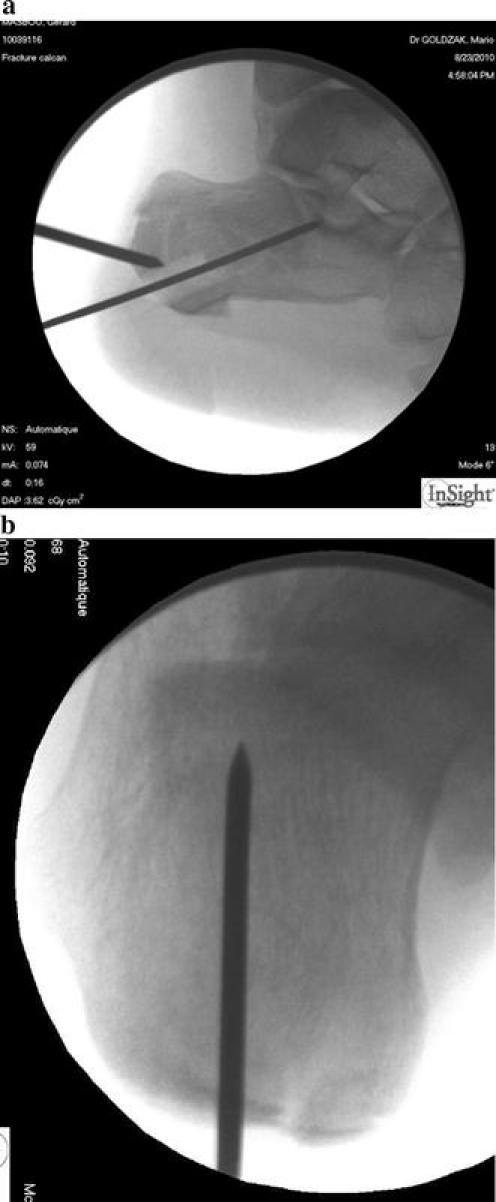
Insertion of the first K wire in the calcaneal tuberosity (**a** lateral view; **b** retrotibial view)

The first step of the procedure is the placement of a K wire in the posterior tuberosity (Fig. 1). In the lateral view, this K wire is placed in the direction of the posterior talar surface, with the same orientation than the posterior bone trabeculae. Using the retrotibial view, the K wire is placed in the middle of the calcaneal tuberosity. The optimal placement follows the direction of the 4th interdigital space.

**Fig. 2:**
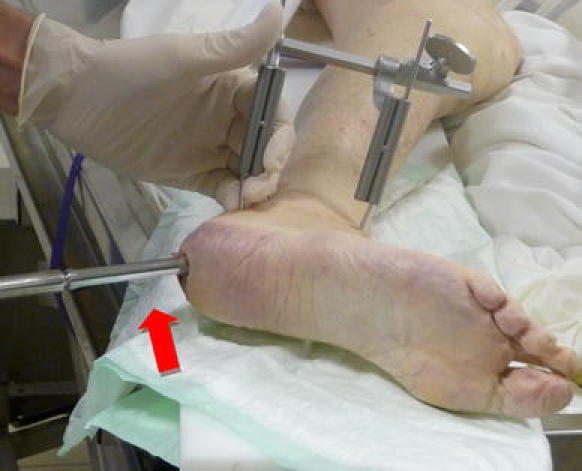
This cadaveric view shows the caspar distractor inserted on the talar and calcaneal pins and the direction of the hollow reamer (*red arrow*) (colour figure online)

Two 3.2 mm pins are then placed laterally, one in the calcaneal posterior tuberosity, the other in the lateral tubercule of the talus (Fig. 2). The K wire inserted in the posterior tuberosity is placed 10 mm above the future tunnel, or inferiorly if the fracture is a tongue-type fracture. The talar K wire is inserted in the lateral tubercule, at the center of the talar dome. A Caspar distractor is then fixed on these wires in order to correct the varus deformity of the great tuberosity and to open the subtalar joint facilitating the reduction of depressed calcaneal surface (Fig. 2).

**Fig. 3:**
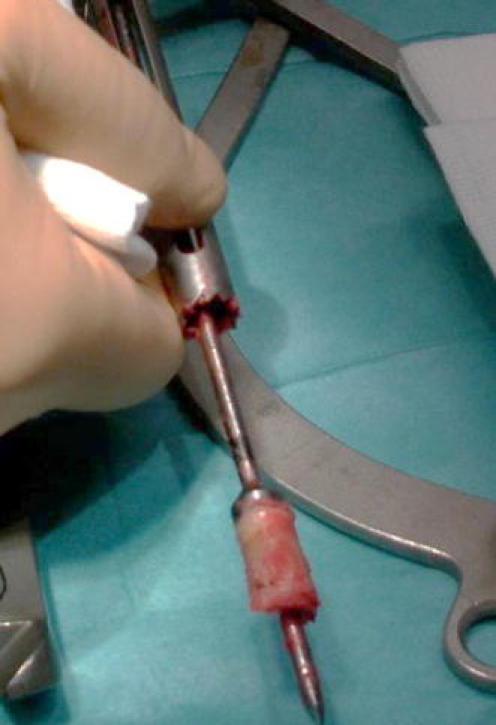
The cylinder of autograft provided by the hollow reamer

The introduction of the 10-mm hollow reamer on the first K wire creates a large tunnel and allows the furniture of a cylindric bone graft (Fig. 3). The hollow reamer has to be directed to the middle of the articular facet, above the Gissane angle. The hollow reamer is then removed and the tunnel protected with a special cup.

**Fig. 4:**
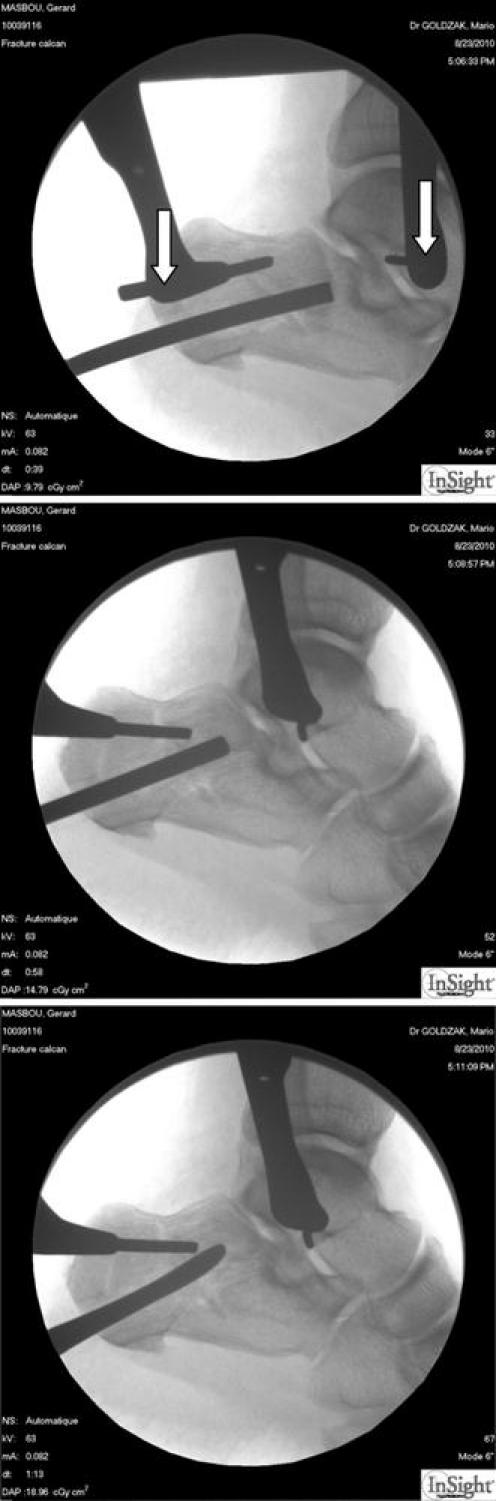
Direct intrafocal reduction by mean of pushers or spatula. *White arrows* show the pins on which the Caspar device is inserted

The next step is the reduction of the depressed articular surface. In the horizontal or vertical displacement with an intact articular surface, it is easy to push upwards the osteochondral fragment with a special bone retractor (Fig. 4). Two dedicated instruments are available, one straight and one hooked. These bone retractors are pushed with a mallet and the progressive reduction checked on the image intensifier. In the case of a mixte fracture with a separation fracture through the articular surface the lateral fragment is vertically displaced and the medial fragment is horizontally depressed. The medial fragment is to be reduced first to restore the height of the calcaneus.

**Fig. 5:**
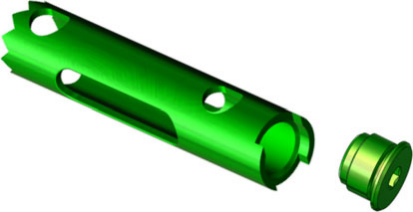
The implant Calcanail. The picture shows the teeth at the tip of the nail, the windows for anchorage of the graft, the holes for locking screws, and the cap

**Fig. 6:**
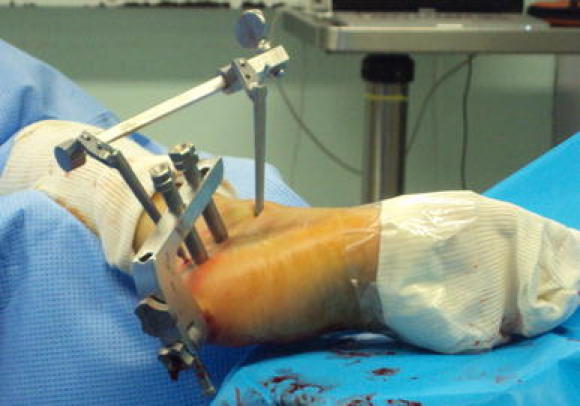
On this operative view are shown the Caspar distractor, the handle of the nail with the sleeves for the insertion of the locking screws

**Fig. 7:**
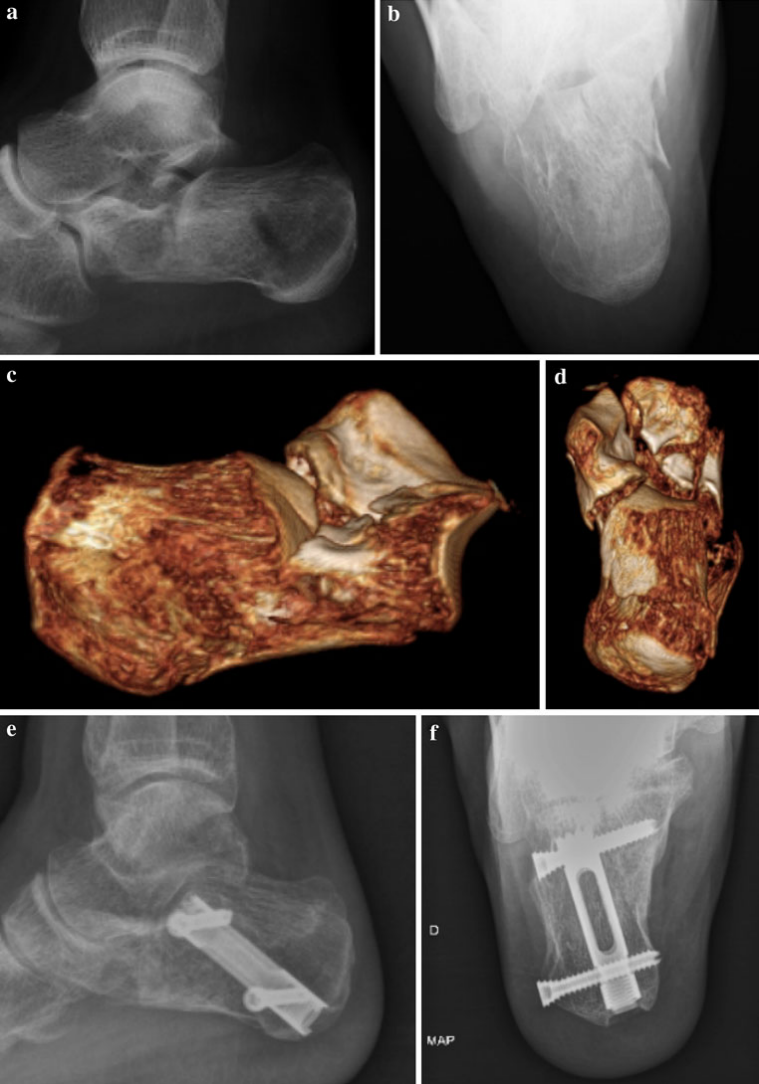
Preoperative and postoperative views of a calcaneal fracture in a 21-year-old woman. **a** Preoperative lateral view, **b** preoperative retrotibial view, **c**, **d** tridimensional preoperative CT, **e** postoperative lateral view, **f** postoperative retrotibial view

The fourth step is the introduction of the nail. This implant is provided in three lengths (45, 50, and 55 mm), one diameter (10 mm), two windows, and two holes for the locking screws (Fig. 5). The indentations at the edge of the nail provide a good anchorage under the subchondral bone. The locking screws are cannulated and introduced on small K wires through special sleeves (Fig. 6). The screws have large heads for compressive effect, and angular stability is achieved through the special shape of the holes of the nail (Fig. 7).

## Discussion

The minimally invasive posterior approach and the innovative reduction technique reduce surgical trauma and complication risk. A good preoperative planning is mandatory: the volume of the depressed fragments and displacements have to be clearly analyzed on the preoperative CT scan. Another advantage of the posterior approach is that the peroneal tendons are protected, impingement avoided, and subtalar mobility preserved.

A preliminary series of 10 cases was reported including four mixtes fractures with two fragments, three mixtes with three articular fragments, two with horizontally displacement and two with vertically displacement [8]. All the cases were operated by the first author. The average AOFAS functional postoperative score was 84/100. The average postoperative Boehler angle was 22° compared to 4° preoperatively. No wound healing problem or infection was noted in this series.

In some multifragmentary fractures, the posterior facet may be so severely damaged that reduction and fixation of the articular surface are not feasible. We consider that a surgical attempt in this case would result in a poor outcome and a primary subtalar arthrodesis may be preferable [9–11]. With this technique, the intraoperative choice between arthrodesis or internal fixation is simple as the approach is the same and the instrumentation for nailing is the same. The arthrodesis implant is 12 mm width. A compressive device fixed on the same two pins than the Caspar distractor may be used for improving compression between the talar and the calcaneal posterior facets.

Specific calcaneal nails are proposed on the market. The Swipp nail is placed horizontally in the lower part of the calcaneus: no direct support of the depressed articular surface is provided by this nail; so additional screws are needed. No publication is available to date [12]. Another nail is proposed, but it just allows the placement of two tuberotalar screws that are inserted through the joint into the talus in order to obtain subtalar arthrodesis through a minimally invasive approach; the technique used to remove cartilage from articular facets is not precised [13]. We consider that currently the proposed implants do not satisfy the expectations for obtaining stability and early postoperative mobilization of the joint. Balloon reduction and cement fixation through percutaneous approach have been proposed but control of the fluid cement in these articular fractures may be difficult [14].

The use of the CALCANAIL in clinical practice offered the following advantages in our experience: (a) the creation of a working channel that provides also a significant bone autograft, (b) the intrafocal reduction of the displaced articular surface in either tongue type or joint depression fractures, (c) the insertion of a locking nail that maintains the reduced articular surface at the right height, (d) the possibility to switch from an ORIF to a reconstruction arthrodesis with the same approach and instrumentation in case of severely damaged posterior facet.

A prospective series started in October 2011, and preliminary results will be published in a future paper.
